# Formation and Detection of Highly Oxidized Hemoglobin Forms in Biological Fluids during Hemolytic Conditions

**DOI:** 10.1155/2020/8929020

**Published:** 2020-04-25

**Authors:** Benard Bogonko Nyakundi, Judit Erdei, Andrea Tóth, Enikő Balogh, Andrea Nagy, Béla Nagy, László Novák, László Bognár, György Paragh, János Kappelmayer, Viktória Jeney

**Affiliations:** ^1^MTA-DE Lendület Vascular Pathophysiology Research Group, Research Centre for Molecular Medicine, Faculty of Medicine, University of Debrecen, 4012 Debrecen, Hungary; ^2^Doctoral School of Molecular Cell and Immune Biology, Faculty of Medicine, University of Debrecen, Debrecen, Hungary; ^3^Department of Pediatrics, Faculty of Medicine, University of Debrecen, Debrecen, Hungary; ^4^Department of Laboratory Medicine, Faculty of Medicine, University of Debrecen, Debrecen, Hungary; ^5^Department of Neurosurgery, Faculty of Medicine, University of Debrecen, Debrecen, Hungary; ^6^Department of Internal Medicine, Faculty of Medicine, University of Debrecen, Debrecen, Hungary

## Abstract

Hemolytic diseases are characterized by an accelerated breakdown of red blood cells (RBCs) and the release of hemoglobin (Hb). Following, RBC lysis Hb oxidation occurs with the formation of different redox states of Hb (metHb and ferrylHb) and the release of heme. ferrylHb is unstable and decomposes to metHb with the concomitant formation of globin radicals and eventually covalently crosslinked Hb multimers. The goal of the present study was to determine the concentrations of the different redox states of Hb in biological samples during hemolytic conditions. We used plasma and urine samples of mice with intravascular hemolysis and human cerebrospinal fluid (CSF) samples following intraventricular hemorrhage. Because ferrylHb is highly unstable, we also addressed the fate of this species. metHb and free heme time-dependently accumulate in plasma and CSF samples following intravascular hemolysis and intraventricular hemorrhage, respectively. ferrylHb is hardly detectable in the biological samples during hemolytic conditions. Under *in vitro* conditions, ferrylHb decomposes quickly to metHb, which process is associated with the formation of covalently crosslinked Hb multimers. We detected these covalently crosslinked Hb multimers in plasma, urine, and CSF samples during hemolytic conditions. Because globin modification is specific for these Hb forms, we propose to call this heterogeneous form of Hb produced during ferrylHb decomposition as globin-modified oxidized Hb (gmoxHb). Understanding the formation and the contribution of gmoxHb species to the pathogenesis of hemolytic conditions could have therapeutic implications in the treatment of hemolytic diseases.

## 1. Introduction

The average life span of red blood cells (RBCs) in the circulation is about 120 days. Earlier destruction of RBCs is defined as hemolysis which can occur in the vasculature or in the extravascular space. Diverse conditions can trigger hemolysis including intrinsic RBC defects such as hemoglobinopathies, RBC enzyme deficiencies and RBC membrane disorders, as well as extrinsic RBC defects triggered by auto- and alloimmune reactions, oxidants, toxins, infections, and mechanical stress (reviewed in [1, 2]).

Upon hemolysis, RBC content is released into the plasma or the extravascular environment. Hemoglobin (Hb), the oxygen carrier molecule, is the far most abundant component of RBCs composing about 96% of the dry weight of RBCs. Hb is a globular tetramer that builds up by 4 subunits, two alpha, and two beta, and each subunits contain a heme prosthetic group in their hydrophobic pocket. Outside of the protective environment of RBCs Hb is prone to oxidation (reviewed in [3]).

Autooxidation of Hb is driven by the Hb-bound molecular oxygen and targets exclusively the ferrous (Fe^2+^) ion of the heme prosthetic group, leading to the formation of superoxide anion and metHb in which the heme group contains a ferric (Fe^3+^) ion in the center [4]. Hydrogen peroxide (H_2_O_2_) and lipid hydroperoxides induce a two-electron oxidation of Hb producing ferrylHb in which the formal charge of heme iron is 4+ [5–7]. Additionally, the reaction of metHb with H_2_O_2_ leads to the formation of ferrylHb globin radical (Hb^•+^(Fe^4+^=O^2-^)) in which the formal charge of heme iron is 4+ and an unpaired electron is located on the globin chain [5–10]. Ferryl iron is considered as a strong oxidant and decomposes quickly via an intramolecular electron transfer between the ferryl iron and specific amino acid residues of the globin chain with close proximity, leading to the further production of globin centered radicals [6, 11–13]. Globin radicals are reactive intermediates which react with each other in which reactions with the unpaired electrons located on the globin chains form new covalent bonds between the Hb subunits yielding covalently crosslinked Hb forms (reviewed in [3]). Importantly, Hb oxidation is associated with remarkable conformational distortion weakening the noncovalent globin-heme interaction, eventually leading to the release of the heme moiety [14–16].

In connection with hemolysis-associated pathologies, most studies focused on the actions of heme. Non-Hb-bound or so-called “free” heme was detected in the plasma and the urine of mice infected by malaria parasites [17, 18]. The contribution of “free” heme to the pathogenesis of malaria was shown by using mouse models of severe malaria with cerebral, renal, and liver manifestations [17–19]. The presence and the pathophysiologic effects of “free” heme have been also established in severe sepsis caused by polymicrobial infections or sickle cell disease caused by mutations in the gene encoding the *β* chain of Hb [20–23]. Heme is a well-known prooxidant, regulates cellular metabolism while exerting proinflammatory and cytotoxic effects [24, 25], but according to a recent study, not all the “free” heme is bioavailable [26]. In fact, its bioavailable concentration under strong hemolytic conditions remains below 5 *μ*mol/L [26].

In contrast, the concentration of acellular Hb and oxidized Hb forms can be particularly high (up to several hundred *μ*mol heme/L) following hemolysis, still the contribution of these Hb forms to the pathogenesis of hemolytic conditions remained rather elusive [27]. The biological effect of Hb depends on the precise nature of heme/iron redox states; therefore, accurate quantification of oxidized Hb forms is particularly important. Recently, more accurate redox-specific extinction coefficients have been determined, allowing precise measurement of ferrous, ferric, and ferryl forms of Hb [28].

The goal of the present study was to determine the concentrations of the different redox states of Hb in biological samples during hemolytic conditions. We used plasma and urine samples of mice with intravascular hemolysis and human cerebrospinal fluid (CSF) samples following intraventricular hemorrhage (IVH). Because ferrylHb is highly unstable, we also aimed at addressing the fate of this reactive species and investigating the formation of covalently crosslinked Hb forms under *in vitro* and *in vivo* conditions. Understanding the formation and the contribution of these species to the pathogenesis of hemolytic conditions could have therapeutic implications in the treatment of hemolytic diseases.

## 2. Methods

### 2.1. Materials

Reagents were purchased from Sigma-Aldrich (St. Louis, MO) unless otherwise specified.

### 2.2. Hemolysis Protocol and Mouse Treatments

Hemolysis was induced in C57BL/6 mice by intraperitoneal (i.p.) administration of phenylhydrazine (PHZ). We applied PHZ twice, first 50 mg/kg body weight and 16 hours later 30 mg/kg body weight. Mice were sacrificed 4 or 16 hours after the first dose of PHZ or 4 hours after the second PHZ injection (20 h). We used age- and sex-matched male and female mice between 6-8 weeks of age. All experiments were carried out in accordance with the principles of the Basel Declaration and followed guidelines of the institutional and national ethical committee and underwent approval (Approval registration number: 2/2016/DEMÁB, issued by the University of Debrecen, Committee of Animal Welfare). Mice were euthanized by CO_2_ inhalation, and blood was drawn by heart puncture into heparinized tubes. Plasma samples were obtained by centrifugation of blood at 2000 × g, 15 min, and 4°C.

### 2.3. Collection of Cerebrospinal Fluid (CSF) Form Patients with IVH

In this work, we used the leftover of CSF samples that were collected by spinal tap or ventricular reservoir puncture for diagnostic purposes at the Department of Neurosurgery, University of Debrecen. CSF samples were taken from preterm infants diagnosed with grade III IVH with a mean gestational age at birth: 27.9 ± 2.2 weeks. CSF samples were collected at 26.6 ± 16.4 days after the onset of IVH. No CSF was obtained exclusively for inclusion in this study. Within 30 minutes of collection, CSF samples were centrifuged (2000 × g, 4°C, and 15 min), and supernatants were stored aliquoted at -70°C until analysis. The procedures were approved by the Scientific and Research Ethics Committee of the University of Debrecen and the Ministry of Human Capacities under the registration number of 1770-5/2018/EÜIG. Parental consent forms were signed by the parents of the infants involved in this study.

### 2.4. Determination of Hb, metHb, ferrylHb, Total Heme, and Free Heme Levels

The absorbance spectra (250-700 nm) of the samples were taken with a spectrophotometer (NanoDrop 2000, Thermo Fisher Scientific, MA, USA). Two methods were used to calculate concentrations of Hb redox forms in biological samples using optical density (OD) values measured at 541, 560, 576, and 630 nm. First, Hb, metHb, and hemichrome concentrations were calculated as described previously by Winterbourn [29]. Second, Hb, metHb, and ferrylHb concentrations were calculated as described previously by Meng and Alayash [28]. Equations used to calculate the concentrations of the different Hb redox forms are listed in Table 1. The total heme concentration of the samples was determined by using a QuantiChrom Heme Assay Kit (Gentaur Ltd., London, UK) according to the manufacturer's instructions. Concentration of non-Hb bound heme in CSF samples was calculated by the following equation: [free heme] = [total heme]–[Hb − heme]–[metHb − heme]–[ferrylhemoglobin]. Concentration of non-Hb bound heme in plasma samples was calculated by the following equation: [free heme] = [total heme]–[Hb − heme]–[metHb − heme]–[hemichrome].

### 2.5. Hemoglobin Preparation

Hb of different redox states, i.e., Hb (Fe^2+^), metHb (Fe^3+^), and ferrylHb (Fe^4+^=O), were prepared as described (Silva et al., [30]). Briefly, Hb was isolated from fresh blood drawn from healthy volunteers using ion-exchange chromatography on a DEAE Sepharose CL-6B column. metHb was generated by incubation (30 min, 25°C) of purified Hb with a 1.5-fold molar excess of K_3_Fe(CN)_6_ over heme. ferrylHb was obtained by incubation (1 h, 37°C) of Hb with a 10 : 1 ratio of H_2_O_2_ to heme. After oxidation, both metHb and ferrylHb were dialyzed against saline (3 times for 3 hours at 4°C) and concentrated using Amicon Ultra centrifugal filter tubes (10,000 MWCO, Millipore Corp., Billerica, MA, USA). Aliquots were snap-frozen in liquid nitrogen and stored at -70°C until use. The purity of each Hb preparation was evaluated by SDS-PAGE followed by staining with ProteoSilver Plus Silver Staining Kit. The purity of Hb preparations was above 99.9%. Molar concentrations for all Hb solutions used throughout this paper are based on heme.

### 2.6. Hemoglobin Oxidation

Hb and metHb at a concentration of 50 *μ*mol/L heme group were oxidized with different amounts of H_2_O_2_ (125, 250, and 500 *μ*mol/L) for 10 minutes at room temperature (RT).

### 2.7. Statistical Analysis

Results are expressed as mean ± SD. At least 3 independent experiments were performed for all in vitro studies. Statistical analyses were performed with GraphPad Prism software (version 8.01, San Diego, CA, USA). Comparisons between more than two groups were carried out by ordinary one-way ANOVA followed by *post hoc* Tukey's multiple comparison test. A value of *p* < 0.05 was considered significant.

## 3. Results

### 3.1. Intravascular Hemolysis and IVH Are Associated with the Formation of Hb Forms with Different Oxidation Status

**Figure 1:**
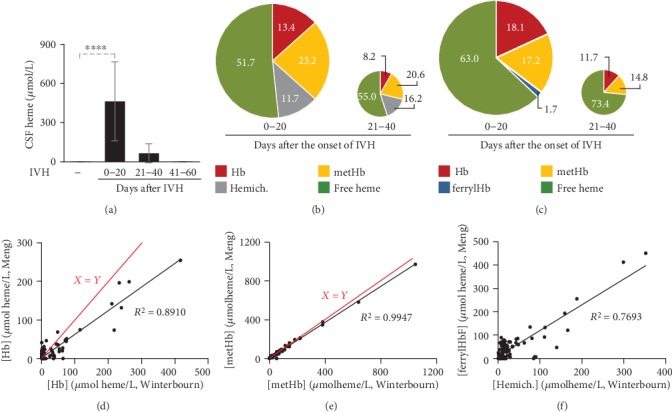
Time-dependent accumulation of heme and different redox states of Hb in biological samples during hemolytic conditions. CSF samples were obtained from preterm infants diagnosed with grade III IVH at different time intervals after the onset of IVH (*n* = 5/group). (a) Total heme levels of CSF were determined by the heme assay kit in triplicates. Bars represent mean ± SD. *p* values were calculated using one-way ANOVA followed by Tukey's multiple comparison analysis. ^∗∗∗^*p* < 0.005, ^∗∗∗∗^*p* < 0.001. (b) Pie chart represents Hb, metHb, and hemichrome levels determined by the method of Winterbourn, and non-Hb-bound heme levels as a percentage of total heme in CSF samples collected between days 0-20 and 21-40 days after the onset of intraventricular hemorrhage. (c) Pie chart represents Hb, metHb, and ferrylHb levels determined by the method of Meng and Alayash, and non-Hb-bound heme levels as a percentage of total heme in CSF samples collected between days 0-20 and 21-40 days after the onset of intraventricular hemorrhage. (d) Scatter plot shows Hb levels of CSF samples (*n* = 114) determined by two different methods of (i) Winterbourn and (ii) Meng and Alayash. Red line represents the *x* = *y* line. (e) Scatter plot shows metHb levels of CSF samples (*n* = 114) determined by two different methods of (i) Winterbourn and (ii) Meng and Alayash. Red line represents the *x* = *y* line. (f) Scatter plot shows the correlation between hemichrome and ferrylHb levels of CSF samples (*n* = 114).

Previous studies showed that Hb tends to be oxidized outside of the protective environment of RBCs in which process Hb species with different oxidation states are formed. In this work, we analyzed human CSF samples following IVH (Figure 1) and murine plasma samples following intravascular hemolysis (Suppl. Fig 1). CSF is a clear, cell-free, plasma-like fluid that occupies the central spinal canal, the ventricular system, and the subarachnoid space. Upon IVH, RBCs enter CSF and lyse. To see whether Hb oxidation occurs in CSF, we used the leftover of CSF samples taken for diagnostic purposes from preterm infants with IVH. In agreement with previous observations, we detected high amounts of extracellular heme (463.01 ± 303.39 *μ*mol/L) in CSF samples obtained between days 0-20 after the onset of intraventricular hemorrhage [31]. Total heme levels were significantly lower in CSF samples obtained at 21-40 days after the onset of hemorrhage (64.98 ± 73.50 *μ*mol/L) and was below 1 *μ*mol/L in CSF samples collected 41-60 days after the bleeding (Figure 1(a)). To see the distribution of the different heme forms in the CSF samples; first, we used the method described by Winterbourn [29] that allowed us to determine Hb, metHb, and hemichrome concentrations in CSF samples (Figure 1(b)). The major heme form in the CSF samples obtained at 0-20 days after the onset of hemorrhage was the non-Hb bound or so-called free heme (51.7 ± 15.7% of total heme, Figure 1(b)). metHb was the second most abundant Hb form in these samples accounting for 23.2 ± 8.2% of total heme content. We also detected hemichrome (11.7 ± 5.3%) and Hb (13.4 ± 4.3%) in these samples (Figure 1(b)). Similarly, free heme and oxidized Hb forms (metHb and hemichrome) were predominant in CSF samples obtained between days 21-40 after the onset of IVH too (Figure 1(b)). Next, we calculated Hb, metHb, and ferrylHb concentrations in the same CSF samples using the method by Meng and Alayash [28]. Similarly to the previously calculated results, we found that free heme is the most abundant heme form (63.0 ± 16.2% of total heme) in the CSF samples obtained between days 0-20 after the onset of hemorrhage (Figure 1(c)). These CSF samples also contained nearly equal amounts of Hb and metHb (18.1 ± 3.2 and 17.2 ± 4.1% of total heme) and a very low amount of ferrylHb (1.7 ± 3.2% of total heme (Figure 1(c)). Free heme becomes even more dominant (73.4 ± 12.3% of total heme) in the expense of the other heme forms in CSF samples obtained between days 21-40 after the onset of intraventricular hemorrhage (Figure 1(d)). To further compare the two methods, we determined Hb and metHb levels of 110 CSF samples with both methods and calculated hemichrome and ferrylHb levels with the appropriate method. We found that the Hb concentrations determined by the two methods correlated strongly (*R*^2^ = 0.8910, Figure 1(d)). At higher Hb concentrations, we measured lower values with the Meng method than with the Winterbourn method (Figure 1(d)). metHb concentrations calculated with the two methods were almost identical (*R*^2^ = 0.9947, Figure 1(e)). Finally, we correlated hemichrome concentrations determined by the method of Winterbourn with the ferrylHb concentration determined by the method of Meng and Alayash (*R*^2^ = 0.7693, Figure 1(f)).

To investigate the formation of oxidized Hb forms in plasma, we induced intravascular hemolysis by intraperitoneal injection of PHZ (50 mg/kg body weight and 30 mg/kg body weight 16 hours later) (Suppl. Fig 1A). We collected plasma samples at 4, 16, and 20 hours after the first PHZ injection. As we expected, PHZ triggered a robust elevation in plasma total heme levels at every time points (Suppl. Fig 1A). Then, we determined the distribution of total plasma heme among Hb, metHb, and hemichrome in the plasma samples of PHZ-injected mice (Suppl. Fig 1B). Plasma samples collected 4 hours after PHZ injection contained mainly native Hb (57.1 ± 12.3% of total heme). In these samples, hemichrome was the second most abundant heme form accounting for 31.2 ± 7.1% of total heme. These samples also contained metHb (11.2 ± 3.1% of total heme) but did not contain free heme (Suppl. Fig 1B). At 16 hours after PHZ injection, the plasma contained a high percentage of hemichrome (46.3 ± 6.2% of total heme), less Hb but more metHb in comparison to the 4-hour time point. Importantly, we could detect non-Hb-bound heme in the plasma samples obtained at 16 hours after PHZ injection (6.6 ± 2.4% of total heme). At 16-hour time point, the mice received a second injection of PHZ that triggered a further increase in total heme, a decrease in the percentage of nonoxidized Hb (13.6 ± 3.6% of total heme), and increased the percentage of non-Hb-bound heme up to 17.5 ± 4.1% of total heme at the 20-hour time point. We could not detect any ferrylHb in plasma samples with the method of Meng and Alayash (data not shown).

Overall, these results suggest that Hb oxidation and heme release occur upon extensive hemolysis (both intravascular and intraventricular). We run into difficulties in detecting ferrylHb in biological samples with visible absorption spectroscopy.

### 3.2. Formation and Detection of Oxidized Hb Forms In Vitro

To study the process and the products of Hb oxidation in detail, we purified Hb from human RBCs. We also produced metHb from Hb by reacting it with K_3_Fe(CN)_6_ that triggers one-electron oxidation of ferrous ion into ferric ion in the heme prosthetic group. The characteristic absorption spectra of Hb and metHb are shown in Supplementary Figure 2A. The purity of the Hb preparations was investigated by silver staining after SDS/PAGE separation. Both Hb and metHb samples gave a single band at 16 kDa that corresponds to the size of an Hb subunit (Suppl. Fig 2B).

**Figure 2:**
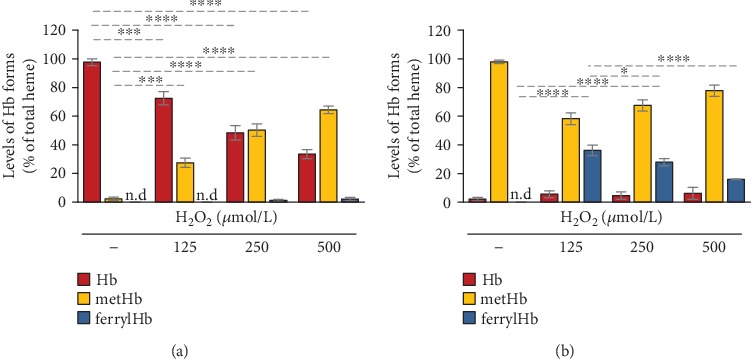
Formation of oxidized Hb forms in hydrogen peroxide-induced oxidation of Hb and metHb. (a) Purified human Hb and (b) metHb (60 *μ*mol/L heme) were oxidized with different concentrations of H_2_O_2_ (125, 250, and 500 *μ*mol/L) for 10 minutes at 37°C. Concentrations of different redox states of Hb were determined by analysis of the visible spectra and the presence of different redox states of Hb as a percentage of total heme was calculated. The bar graph shows mean ± SD from 3 independent experiments. *p* values were calculated using one-way ANOVA followed by Tukey's multiple comparison analysis. ^∗^*p* < 0.05, ^∗∗∗^*p* < 0.005, ^∗∗∗∗^*p* < 0.001.

To induce oxidation, we reacted Hb and metHb with H_2_O_2_ and take absorption spectra of the samples and calculated the concentrations of Hb, metHb, and ferrylHb with the use of the extinction coefficients and equations determined recently by Meng and Alayash [28]. Reacting Hb (50 *μ*mol/L) with increasing concentrations of H_2_O_2_ (125-500 *μ*mol/L) for 10 minutes at RT triggered a dose-dependent formation of metHb, and parallel with that, a decrease of Hb concentration (Figure 2(a)). We observed ferrylHb formation (~1% of total heme content) when Hb was reacted with high doses (250 and 500 *μ*mol/L) of H_2_O_2_ (Figure 2(a)). Next, we reacted metHb with H_2_O_2_ (125-500 *μ*mol/L), and we could detect ferrylHb 10 minutes after the reaction started (Figure 2(b)). We found that after a reaction in which Hb was reacted with a 2.5-fold molar excess of H_2_O_2_ (125 *μ*mol/L), 36.18 ± 3.62% of total Hb was in the ferryl form (Figure 2(b)). Unexpectedly, when we applied H_2_O_2_ in a 10-fold molar excess (500 *μ*mol/L) over Hb, we obtained a significantly lower yield of ferrylHb formation (16.0 ± 0.23% of total heme) (Figure 2(b)).

**Figure 3:**
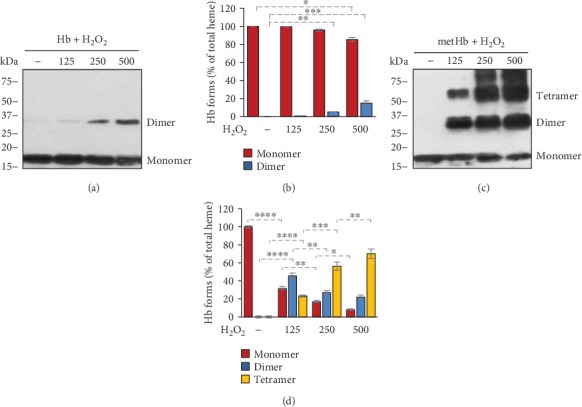
Formation of covalently crosslinked Hb multimers in hydrogen peroxide-induced oxidation of Hb and metHb. Purified human Hb and metHb (60 *μ*mol/L heme) were oxidized with different concentrations of H_2_O_2_ (125, 250, and 500 *μ*mol/L) for 30 minutes at 37°C. (a, c) Representative western blots after (a) Hb and (c) metHb oxidation. (b, d) Densitometric analysis of western blots was performed and the percentages of gmoxHb multimers as a percent of total Hb were calculated. The bar graph shows mean ± SD from 3 independent experiments. *p* values were calculated using one-way ANOVA followed by Tukey's multiple comparison analysis. ^∗^*p* < 0.05, ^∗∗^*p* < 0.01, ^∗∗∗^*p* < 0.005, ^∗∗∗∗^*p* < 0.001.

The ferryl (Fe^4+^) oxidation state is highly unstable. Supporting this notion, it has been shown that the ferryl ion in ferrylHb readily oxidizes particular oxidation-prone adjacent amino acid residues of the globin chain, resulting in the formation of globin radicals and ferric (Fe^3+^) ion. The produced globin radicals are also reactive and can stabilize by reacting with each other. In the reaction between the globin radicals, a new covalent bond is formed from the two unpaired electrons located on the globin radicals, resulting in terminally crosslinked Hb multimers. Therefore, next, we wanted to examine whether this reaction occurred in our experimental system. We incubated Hb and metHb (50 *μ*mol/L) with different concentrations of H_2_O_2_ (125-500 *μ*mol/L) for 10 minutes at RT and subjected the samples to denaturing SDS-PAGE followed by Western blot to detected Hb. We observed dose-dependent formation of covalently crosslinked Hb dimers in the reaction between Hb and H_2_O_2_ (Figures 3(a) and 3(b)). Moreover, we found a large exacerbation of Hb dimer formation in the reaction between metHb and H_2_O_2_ (Figures 3(c) and 3(d)). Besides dimers, we observed a dose-dependent formation of tetramers when metHb was reacted with H_2_O_2_ (Figures 3(c) and 3(d)). Because globin modification is specific for these covalently crosslinked Hb multimers, we propose to call this heterogeneous form of Hb produced during ferrylHb decomposition as globin modified oxidized Hb (gmoxHb).

**Figure 4:**
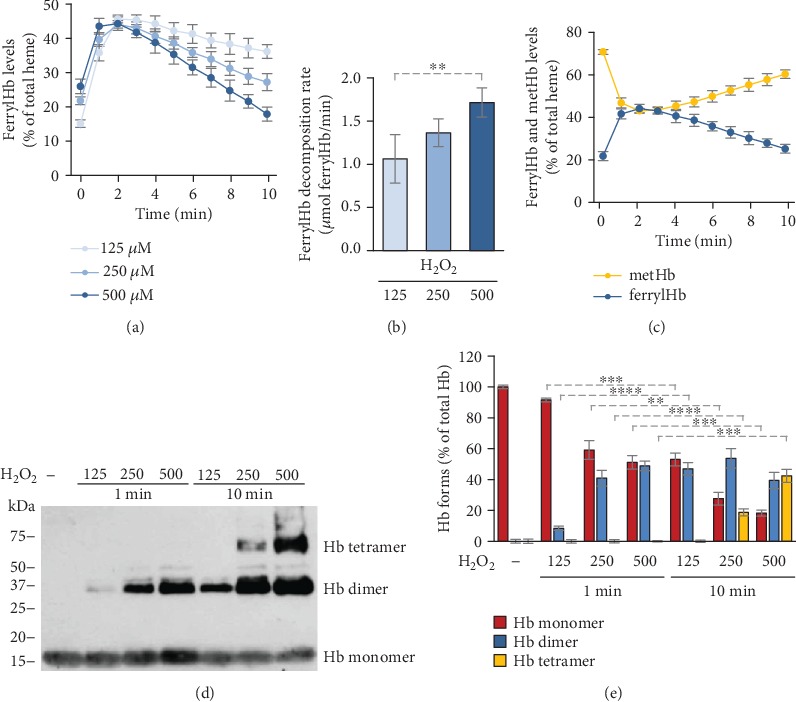
Decay of ferrylHb leads to the regeneration of metHb and formation of gmoxHb multimers. (a–c) Purified human metHb (60 *μ*mol/L heme) was oxidized with different concentrations of H_2_O_2_ (125, 250, and 500 *μ*mol/L), and the concentrations of different redox states of Hb were determined every minute for 10 minutes. (a) Time- and dose-dependent formation and decomposition of ferrylHb in the reaction between metHb and H_2_O_2_ presented as mean ± SD of 3 independent experiments. (b) ferrylHb decomposition rate (*μ*mol ferrylHb/min) was calculated at each H_2_O_2_ concentration from the kinetic measurements. Graph shows mean ± SD of 3 independent experiments. (c) Time-dependent inverse changes of ferrylHb and metHb levels in the course of metHb oxidation with H_2_O_2_ (500 *μ*mol/L) presented as mean ± SD of 3 independent experiments. (d, e) Time- and dose-dependent formation of gmoxHb in the course of metHb oxidation with H_2_O_2_. Purified human metHb (60 *μ*mol/L heme) was oxidized with different concentrations of H_2_O_2_ (125, 250, and 500 *μ*mol/L) for 1 and 10 minutes at 37°C. (d) Representative western blot is shown. (e) Densitometric analysis of western blots was performed, and the percentages of gmoxHb monomers, dimers, and tetramers as a percent of total Hb were calculated. The bar graph shows mean ± SD from 3 independent experiments. *p* values were calculated using one-way ANOVA followed by Tukey's multiple comparison analysis. ^∗∗^*p* < 0.01, ^∗∗∗^*p* < 0.005, ^∗∗∗∗^*p* < 0.001.

Next, we measured the kinetics of ferrylHb production and decomposition using similar experimental settings as in our previous experiment. We mixed metHb (50 *μ*mol/L) with H_2_O_2_ at different concentrations (125-500 *μ*mol/L), followed the reaction by taking absorption spectra of the samples in every minute for 10 minutes, and calculated the concentrations of ferrylHb and metHb. ferrylHb level peaked at around 2 minutes after the reaction was started and then declined during the 10-minute examination period at all H_2_O_2_ concentrations (Figure 4(a)). The peak level of ferrylHb (~45% of total Hb) was independent of the concentration of H_2_O_2_ used in the reaction (Figure 4(a)). On the other hand, the rate of ferrylHb decomposition was dependent on the dose of H_2_O_2_. Namely, the highest concentration of H_2_O_2_ (500 *μ*mol/L) triggered the highest rate of ferrylHb disappearance (1.72 ± 0.17 *μ*mol/L ferrylHb/min) (Figures 4(a) and 4(b)). As we expected, metHb and ferrylHb levels changed the exact opposite directions in the course of the reaction between metHb and H_2_O_2_ (Figure 4(c)). Next, we investigated the concentration- and time-dependence of the formation of gmoxHb. We observed that H_2_O_2_ dose-dependently triggered the formation of gmoxHb dimers within 1 minute (Figures 4(d) and 4(e)). Moreover, 250 and 500 *μ*mol/L H_2_O_2_ induced the formation of gmoxHb tetramers within 10 minutes in a dose-dependent manner (Figures 4(d) and 4(e)).

### 3.3. Detection of gmoxHb Forms in Biological Samples

**Figure 5:**
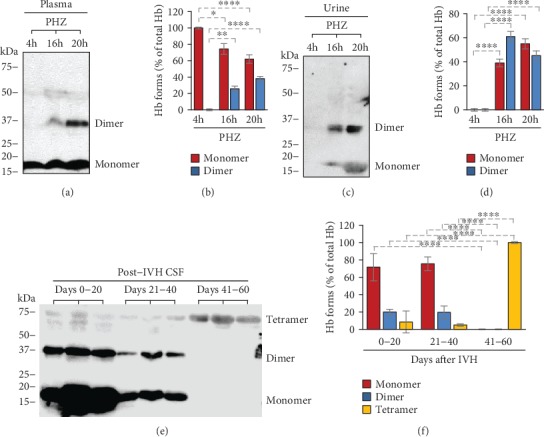
Time-dependent accumulation of covalently crosslinked Hb multimers in biological samples under hemolytic conditions. (a–d) Plasma and urine samples were collected from C57BL/6 mice at different time points (*n* = 5/group) following PHZ injection. (a, c) Representative western blots and (b, d) densitometric analysis of western blots are presented. The bar graph shows mean ± SD from 3 independent experiments. (e, f) CSF samples were obtained from preterm infants diagnosed with grade III IVH at different time intervals after the onset of IVH (*n* = 5/group). (e) Representative western blot is shown. Densitometric analysis was performed and percentages of Hb monomers, dimers, and tetramers as a percent of total Hb were calculated. *p* values were calculated using one-way ANOVA followed by Tukey's multiple comparison analysis. ^∗^*p* < 0.05, ^∗∗^*p* < 0.01, ^∗∗∗^*p* < 0.005, ^∗∗∗∗^*p* < 0.001.

Finally, we investigated whether these covalently crosslinked Hb forms are present in different biological samples. First, we analyzed mice plasma and urine samples following intravascular hemolysis. Intravascular hemolysis was induced with intraperitoneal injection of PHZ as described previously. Plasma and urine samples were collected at 4, 16, and 20 hours after the PHZ injection. Four hours after the PHZ injection, the plasma contained only Hb monomers, and we could not detect any gmoxHb in the urine (Figures 5(a)–5(d)). In contrast, all the plasma and urine samples obtained at 16 and 20 hours post-PHZ injection contained gmoxHb dimers (Figures 5(a)–5(d)). Besides plasma and urine, we also investigated the presence of gmoxHb forms in human CSF samples following IVH. We found gmoxHb dimers (~20% of total heme) in CSF samples collected between days 0-20 and 21-40 after the onset of IVH (Figure 5(f)). Interestingly, we found exclusively gmoxHb tetramers in CSF samples collected at day 41-60 post-IVH (Figure 5(f)).

## 4. Discussion

Despite the heterogeneous etiology of hemolytic diseases, there are common symptoms including (i) endothelial activation/dysfunction responsible for hypercoagulation state and, eventually, thrombus formation; (ii) reduced nitric oxide bioavailability leading to vasoconstriction, pulmonary, and systemic hypertension; and (iii) hemoglobinuria and acute kidney injury [2]. These common symptoms of hemolytic diseases are largely induced by extracellular Hb and its breakdown products.

During hemolysis, Hb is released from RBCs and is bound avidly by haptoglobin (Hp), an acute phase plasma protein that protects Hb from oxidation and facilitates its clearance from the circulation through endocytosis via the CD163 macrophage scavenger receptor [32–38]. The plasma concentration of Hp is relatively high (0.41–1.65 mg/ml), allowing the elimination of approximately 3 g of Hb which is less than 1% of the circulating Hb. Upon massive hemolysis, the Hb scavenging capacity of Hp is overwhelmed and Hb accumulates in the plasma. Extracellular Hb scavenges nitric oxide, the important vasodilator (reviewed in [39, 40]) leading to vasoconstriction and hypertension [41]. Furthermore, noncompartmentalized Hb cannot benefit from the highly efficient antioxidant defense system present in intact RBCs, and Hb tends to oxidize [3].

In this study, we investigated the formation of different redox states of Hb in biological samples during hemolytic conditions. First, we analyzed CSF samples obtained from premature infants following IVH. IVH is the bleeding into the brain's ventricular system that is filled up with CSF. It is a frequent complication of prematurity that associates with high neonatal mortality and an increased risk of neurodevelopmental impairment in the surviving infants [42–45]. It is long known that inflammation plays a critical role in the pathophysiology of IVH-induced brain damage; however, the molecular mechanism by which IVH stimulates the inflammatory response is not fully understood. Extravasation of blood into the intraventricular space triggers a cascade of events including the release of various vasoactive and proinflammatory molecules from the blood (reviewed in [46]). Previous studies showed that cell-free Hb and Hb metabolites are present in CSF following different types of intracranial hemorrhage including IVH [31, 47–49]. Among the different redox states of Hb, metHb was detected in CSF samples obtained following IVH in preterm infants as well as in an experimental rabbit model of IVH and its role in the neuroinflammatory response has been shown [47]. In agreement with these previous observations, we found that after IVH Hb is released into the CSF and Hb oxidation occurs leading to the formation of metHb and non-Hb-bound heme in a time-dependent manner. In this study, we compared two different visible spectrophotometric methods to calculate the concentrations of the different redox states of Hb. We used the method of Winterbourn [29] to calculate Hb, metHb, and hemichrome concentrations, whereas the method of Meng and Alayash [28] was used to calculate Hb, metHb, and ferrylHb concentrations. Hb concentrations measured by the two methods correlated well, and metHb concentrations were almost identical. The Winterbourn method allowed us to detect hemichrome in the CSF samples, whereas the other method allowed us to detect—although low concentration—ferrylHb in the CSF samples. These oxidized Hb forms might play distinct roles in the neuroinflammatory response following IVH.

Besides CSF analysis, we used a well-established model of acute sterile hemolysis to investigate the formation of the different Hb species *in vivo*. In agreement with previous observations [27, 50, 51], we found that the injection of PHZ triggered a marked elevation of extracellular heme content in the plasma. The determination of the distribution of the extracellular heme among the different redox states of Hb revealed that Hb dominates at an early time point (4 h), and hemichrome becomes more dominant at later time points. Previous studies showed that hemichrome formation is a characteristic feature of PHZ-induced hemolysis, as PHZ reacts with Hb, forming reactive intermediates such as a hydroxyl radical which triggers the production of hemichrome [52]. On the other hand, we could not detect ferrylHb in plasma samples after PHZ injection. This might be due to the transient nature of ferrylHb or the inadequacy of the method to measure ferrylHb in such a complex biological sample. Nevertheless, this question needs to be further investigated.

PHZ-induced intravascular hemolysis is associated with acute kidney injury, due to oxidative stress, cytotoxicity, and proinflammatory effects triggered by extracellular Hb and its breakdown products. According to a recent study, the renal manifestations of intravascular hemolysis are largely heme-independent, since injection of free heme could not reproduce them, and heme scavenging could not prevent them, which suggests that kidney injury is mediated by hemolysis-derived products upstream of heme release [51]. We assume that oxidized Hb forms could play a role in hemolysis-induced kidney injury, which assumption should be addressed and the exact Hb forms should be identified in further studies.

The autooxidation of Hb leads to the formation of metHb and superoxide anion [5–7]. Hydrogen peroxide is also produced during Hb autoxidation by the spontaneous or enzyme-driven dismutation of superoxide [53]. Hydrogen peroxide initiates a two-electron oxidation of Hb leading to the formation of ferrylHb [5–7]. Interestingly, we could hardly detect any ferrylHb in our biological samples. We used a recently published spectrophotometric method to measure the different redox states of Hb in the plasma and CSF samples [28]. The unsuccessful detection of ferrylHb in biological samples might be due to the unstable nature of this high valance redox state of heme iron in ferrylHb.

Using *in vitro* approaches, we investigated the stability and the fate of ferrylHb during hydrogen peroxide-induced Hb and metHb oxidation. In the reaction between metHb and hydrogen peroxide paradoxically, we observed that the formation of ferrylHb was inversely proportional to the concentration of hydrogen peroxide. Further investigation of the kinetics of the reaction between metHb and hydrogen peroxide revealed that ferrylHb concentration reaches its maximum at around two minutes after initiation of the reaction, and then, ferrylHb decomposes with a rate that is dependent on the concentration of hydrogen peroxide. In line with this notion, higher concentrations of hydrogen peroxide triggered a faster decomposition rate of ferrylHb resulting in lower levels of ferrylHb measured at 10 minutes after the initiation of the reaction.

Based on the literature, ferrylHb decomposition occurs via an intramolecular electron transfer between the ferryl iron and specific amino acid residues of the globin chain, leading to the production of ferric iron and globin centered radicals [6, 11–13]. The reaction between globin radicals results in covalently crosslinked Hb multimers (reviewed in [3]). Here, we showed that metHb and ferrylHb levels are inversely related to each other in the course of the reaction between metHb and hydrogen peroxide. Moreover, we found time- and dose-dependent production of covalently crosslinked Hb multimers.

The scientific literature lacks a consensus regarding the nomenclature of this covalently crosslinked Hb multimers. The formation of covalently crosslinked Hb multimers is ultimately linked to two-electron oxidation of Hb or metHb. The heme iron in these forms is in the ferric redox state; therefore, in many works, these forms are referred simply as metHb. We believe that this nomenclature is not correct because it overlooks the modification of the globin moiety that gives this Hb form unique properties. In other works, authors refer these forms as ferrylHb that is confusing as well, because the ferryl oxidation state is temporary. Because globin modification is a major characteristic feature of this Hb form, in this work, we proposed to call these covalently crosslinked Hb forms as gmoxHb. A unique name could make it easier to distinguish these species from metHb that is an oxidized Hb without globin modification and from ferrylHb that is an unstable redox form of Hb.

As we stated before, gmoxHb has unique biological properties that distinguish it clearly from Hb and metHb. It has been previously shown that gmoxHb acts in a proinflammatory manner targeting endothelial cells and macrophages independently of heme release. The exposure of endothelial cells to gmoxHb induces the upregulation of adhesion molecules such as vascular cell adhesion molecule-1, intercellular adhesion molecule-1, and E-selectin and induces intercellular gap formation that is dependent on actin polymerization and the activation of the c-Jun N-terminal kinase and the p38 mitogen-activated protein kinase signal transduction pathways [3, 30]. Moreover, gmoxHb serves as a damage-associated molecular pattern (DAMP) inducing the Nucleotide-binding domain, Leucine-rich Repeat containing Protein 3 (NLRP3) inflammasome activation, and subsequent production of interleukin 1 beta in macrophages [27].

Although we could hardly detect ferrylHb in the plasma and CSF samples after intravascular hemolysis and IVH, respectively, we hypothesized that ferrylHb formed but decomposed quickly making the detection of this temporary product impossible. We found that indeed this was the case and detected high amounts of gmoxHb dimers in both plasma and urine samples following intravascular hemolysis.

Both Hb globin chains contain several oxidation-prone amino acid residues with close proximity to the heme iron (i.e., 𝛼Tyr-24, 𝛼Tyr-42, 𝛼His-20, 𝛽Tyr-35, 𝛽Tyr-130, and 𝛽Cys-93) which can be involved in the intramolecular electron transfer between the ferryl iron and the globin [3, 12, 13]. Because of this phenomenon, not only dimers but higher multimers can be formed during Hb oxidation. Along with this notion, we detected the time- and dose-dependent formation of gmoxHb tetramers when metHb was reacted with H_2_O_2_*in vitro*. Interestingly, we could not detect gmoxHb tetramers in plasma or urine samples following intravascular hemolysis, but this form was present in CSF samples obtained from premature infants following IVH. In fact, gmoxHb was the exclusively represented Hb form in CSF samples collected between days 41-60 posthemorrhage.

CSF is renewed 4-5 times a day via the blood-CSF barrier [54]. Specific proteins expressed in the choroid plexus epithelial cells tightly regulate the transport of essential nutrients and ions into, and removal of waste products from the CNS via diffusion, facilitated diffusion and active transport [55]. We lack information about whether Hb and its derivatives can be cleared from CSF via the blood-CSF barrier following IVH, but our results do not support this idea. We found that Hb monomers are present in the CSF up to 40 days post-IVH which suggests a slow clearance mechanism for Hb.

Hp, the Hb binding protein, plays a crucial role in removing extracellular Hb from plasma. Upon massive intravascular hemolysis, the Hb removal capacity of plasma is overwhelmed, and Hb can be excreted in the urine, which occurred in our PHZ-injected mice model. This pathological event can cause acute kidney injury [56], but at the same time it prevents the long-lasting presence of cell-free Hb in the circulation.

Hp is present in CSF too, but its concentration is much lower than in the plasma, therefore, the Hb-binding capacity of CSF (~100 *μ*g Hb in adults, no data about infants) is far below the Hb-binding capacity of plasma (~5 g Hb) [57]. We showed earlier that CSF samples obtained between days 0-20 after the onset of hemorrhage contained 10-530 mg of Hb in the 50 ml volume of CSF, and assumed that in most cases of grade III IVH, the level of cell-free Hb exceeds the Hb-binding capacity of CSF [31]. This assumption is supported by the observation that following IVH, Hb penetrates from the intraventricular space to the periventricular white matter and contributes to the development of IVH-associated brain injury [58–60]. Besides Hb clearance, Hp plays a role in protecting Hb from oxidation, by shielding its oxidation-prone amino acid residues [61]. This mechanism could have limited function upon intraventricular hemorrhage due to the low concentration of Hp in CSF. Taken together, low Hp concentration in CSF allows the long-lasting presence and extensive oxidation of Hb in the CSF which eventually leads to the formation of gmoxHb tetramers. We assume that the clearance mechanisms—if any—are limited in the case of gmoxHb tetramers that could explain the exclusive presence of this Hb form in the CSF samples collected between days 41-60 after the onset of IVH. Nevertheless, further investigations are needed to address the clearance mechanisms of Hb and its derivatives from the CSF after IVH.

GmoxHb crosslinks have been detected in human complicated atherosclerotic lesions and their etiopathogenetic role in oxidizing low-density lipoprotein has been established previously [9, 10, 62, 63]. Further studies needed to understand the role of gmoxHb in the pathogenesis of hemolytic diseases, and because gmoxHb is a heterogeneous entity, the particular roles of the Hb crosslinks with different sizes should be addressed.

## Figures and Tables

**Table 1:** 

Calculations of Hb redox forms (mmol heme group/L)	Ref.
[Hb] = −350.52 × OD_541_ + 388.95 × OD_576_ + 150.02 × OD_630_ [metHb] = −185.77 × OD_541_ + 171.88 × OD_576_ + 387.58 × OD_630_ [ferrylHb] = 702.23 × OD_541_ − 657.43 × OD_576_–455.64 × OD_630_	[28]
[Hb] = 119 × OD_576_ − 39 × OD_630_ − 89 × OD_560_ [metHb] = 28 × OD_576_ + 307 × OD_630_ − 55 × OD_560_ [hemichrome] = −133 × OD_576_ − 114 × OD_630_ + 233 × OD_560_	[29]

## Data Availability

The authors confirm that the data supporting the findings of this study are available within the article.

## Abbreviations

CD163: Cluster of differentiation 163
CSF: Cerebrospinal fluid
DEAE: Diethyl-amino-ethyl
ferrylHb: Ferryl hemoglobin
gmoxHb: Globin-modified oxidized Hb
H_2_O_2_: Hydrogen peroxide
Hb: Hemoglobin
IVH: Intraventricular hemorrhage
K_3_Fe(CN)_6_: Potassium ferricyanide
metHb: Methemoglobin
PHZ: Phenylhydrazine
RBCs: Red blood cells
SDS-PAGE: Sodium dodecyl sulfate-polyacrylamide gel electrophoresis
OD: Optical density.

## Supplementary Materials

Supplementary Materials Supplementary Figure 1: time-dependent accumulation of heme in plasma of PHZ-injected mice. Supplementary 2. Supplementary Figure 2: characterization of purified human Hb and metHb solutions.
