# Supplementary material for: Formation and Detection of Highly Oxidized Hemoglobin Forms in Biological Fluids during Hemolytic Conditions
Source: Oxid Med Cell Longev. 2020 Apr 25;2020:8929020. doi: 10.1155/2020/8929020 (PMC7196973; doi:10.1155/2020/8929020)
Supplement: Supplementary Materials — Supplementary Figure 1: time-dependent accumulation of heme in plasma of PHZ-injected mice. Supplementary 2. Supplementary Figure 2: characterization of purified human Hb and metHb solutions. [file 8929020.f1.pdf]

SUPPLEMENTARY MATERIAL

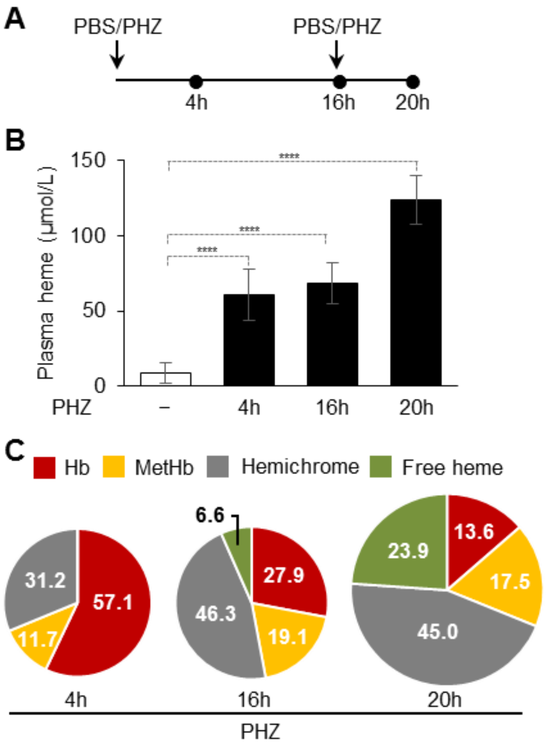

Supplementary Figure 1.
Time-dependent accumulation of heme in plasma of PHZ-injected mice
(A) C57BL/6 mice were injected (i.p.) with PHZ (50mg/kg, then 30 mg/kg mice at 16h, n=5) or PBS (n=5). Mice were sacrificed and plasma samples were collected at 4, 16 and 20h time points. (A) Schedule of the experiment. (B) Total heme levels of plasma samples were determined by heme assay kit in triplicates. Bars represent mean ± SD. P values were calculated using one-way ANOVA followed by Tukey's multiple comparison analysis. ****$p<0.001$. (C) Presence of different redox states of Hb (Hb, metHb, hemichrome) and non Hb-bound heme as a percentage of total heme in plasma at different time points following PHZ injection is shown by pie charts.

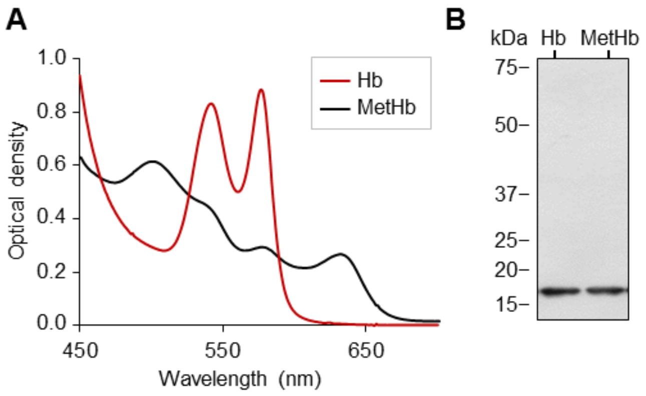

Supplementary Figure 2.

Characterization of purified human Hb and metHb solutions

(A) Absorbance spectra of purified Hb and metHb solutions at a concentrations of 60 μmol/L. Optical densities (OD) at 541, 576 and 630 nm were used to calculate the concentrations of Hb and metHb using the following equations: $[Hb] = -350.52 \times OD_{541} + 388.95 \times OD_{576} + 150.02 \times OD_{630}$, $[metHb] = -185.77 \times OD_{541} + 171.88 \times OD_{576} + 387.58 \times OD_{630}$. (B) A representative western blot of Hb and metHb (5 nmol/lane) is depicted.
